# Serum Krebs von den Lungen-6 for Predicting the Severity of COVID-19 Lung Injury: A Systematic Review and Meta-Analysis

**DOI:** 10.52547/ibj.25.6.381

**Published:** 2021-10-06

**Authors:** Andro Pramana Witarto, Bendix Samarta Witarto, Achmad Januar Er Putra, Shidi Laras Pramudito, Alfian Nur Rosyid

**Affiliations:** 1 Medical Program, Faculty of Medicine, Universitas Airlangga, Indonesia;; 2 Department of Pulmonology and Respiratory Medicine, Universitas Airlangga Hospital, Faculty of Medicine, Universitas Airlangga, Indonesia

**Keywords:** Biomarker, COVID-19, Infectious disease, Krebs von den Lungen-6, Lung injury

## Abstract

**Introduction:**

Lung injury is common in COVID-19 patients. The severity of lung injury appears to be reflected in serum KL-6, a glycoprotein expressed on type II alveolar epithelium. This study aims to assess the role of serum KL-6 in reflecting the severity of lung injury in COVID-19 patients.

**Methods:**

A systematic search was conducted in Scopus, PubMed, Wiley Online Library, and ProQuest. Articles were screened based on several eligibility criteria and assessed for study quality using NOS.

**Results:**

This systematic review included four studies involving a total of 151 adult COVID-19 patients. Pooled analysis revealed that serum KL-6 was significantly higher in severe patients (SMD = 1.16; 95% CI = 0.69–1.63) with moderately high pooled sensitivity (79%; 95% CI = 61–91%) and specificity (86%; 95% CI = 72–95%).

**Conclusion:**

High serum KL-6 may depict more severe lung injury in COVID-19 patients with moderately high sensitivity and specificity.

## INTRODUCTION

COVID-19 is a disease caused by SARS-CoV-2^[^^1^^]^. It is widely known that the virus gains access to the cell through the angiotensin-converting enzyme-2 receptor^[^^2^^]^. According to the World Health Organization, COVID-19 is divided into three categories: suspected case, probable case, and confirmed case^[^^3^^]^. The disease severity is further divided into asymptomatic, mild, moderate (pneumonia), severe (severe pneumonia), and critical (acute respiratory distress syndrome, sepsis, and septic shock)^[^^1^^]^. ARDS is the most common clinical presentation in moderate, severe, and critical COVID-19 patients. This syndrome is also presented as the manifestation of COVID-19 lung injury due to diffuse damage to the alveolar cells^[^^4^^]^. 

The severity of lung injury in certain diseases or conditions may be reflected in the serum KL-6, a glycoprotein mainly expressed on type II alveolar epithelium cells^[^^5^^]^. Its role in lung diseases has been observed in rheumatoid-related interstitial lung disease, idiopathic interstitial pneumonia, acute exacerbation in idiopathic pulmonary fibrosis, lung cancer, and connective tissue-related interstitial lung disease^[^^5^^-^^9^^]^. Some studies have also shown that the severity of COVID-19 patients is reflected through the higher KL-6 levels in the blood, thus indicating type II pneumocyte damage and lung injury^[^^10^^,^^11^^]^. However, to date, no study has defined and summarized the role of KL-6 as a novel biomarker in predicting COVID-19 severity, including its sensitivity and specificity in confirming its function. Therefore, in this study, we conducted a systematic review and meta-analysis to evaluate and clarify the role of KL-6 in determining COVID -19 severity.

## MATERIALS AND METHODS

This systematic review was conducted based on PRISMA statement^[^^12^^]^. A detailed protocol of this study has previously been registered in PROSPERO (CRD42021234457).

**Data search strategy**

Computerized data searching was conducted independently by all authors in four databases, including Scopus, PubMed, Wiley Online Library, and ProQuest. Relevant studies were retrieved from inception to 22 November 2020 with keywords based on Medical Subject Headings (MeSH) terms, and other additional terms listed as follows: ((“COVID-19”) OR (“COVID19”) OR (“Sars-CoV-2 infection”) OR (“2019-nCoV infection”) OR (“coronavirus disease 2019”)) AND ((“KL-6”) OR (“Krebs von den Lungen-6”)) AND ((“severe”) OR (“severity”)). The search was limited to human participants without any language restriction.

**Eligibility criteria**

The inclusion criteria of this study were as follows: (1) observational study, (2) study population consisted of adult patients (>18 years old) with a confirmed diagnosis of COVID-19 and was classified according to the disease severity, and (3) the measured outcomes were comparing serum KL-6 levels among the study groups. However, the exclusion criteria included studies with irrelevant titles and abstracts, irretrievable full-text articles, non-English studies, review articles, case reports, case series, and conference abstracts.

**Data synthesis and quality assessment**

Four investigators (AP, BS, AJ, and SL) screened the literature independently. Any disagreements were resolved in a consensus involving all authors. The extracted data were based on author and publication year, the case definition of COVID-19 and its classification, study location, study design, study population, sample size, age of patients, serum KL-6 levels, and study outcomes as expressed by *p *value in each study with receiver operating characteristics analysis, best cut-off value, sensitivity value, and specificity value if available. The quality assessment of the selected studies was performed using the NOS tool to evaluate the risk of bias of each study. NOS interpretation in the cross-sectional study is classified into a very good study (score 9-10), good study (score 7-8), satisfactory study (score 5-6), and unsatisfactory study (score 0-4), while in the cohort study is classified into a good-quality study (score 7-9), moderate-quality study (score 4-6), and poor-quality study (score 0-3). The quality assessment was conducted by two reviewers (AP and BS) collaboratively through a group discussion, and the final decision was taken based on the agreement of both reviewers. 

**Statistical analysis**

AP and BS performed a meta-analysis of mean difference using RevMan 5.4.To perform meta-analysis, the median and IQR data from the included studies were transformed into mean and SD using a standardized online calculator (http://www.math.hkbu. edu.hk/~tongt/papers/median2mean.html)^[^^13^^]^. Pooled analyses of sensitivity, specificity, +LR, -LR, and DOR were also performed using Meta-DiSc 1.4^[^^14^^]^ by constructing a 2 × 2 table for studies with sufficient data. However, the area under the summary receiver operating characteristic curve was not determined due to the small number of studies. Heterogeneity between studies was assessed with a chi-square test (Cochran’s Q statistic) and quantified with the Higgins’ *I*^2^ statistic. The level of heterogeneity was determined using *I*^2^ values. *I*^2 ^< 25% was considered as low heterogeneity, 25%–75% as moderate heterogeneity, and *I*^2^ > 75% as high heterogeneity. A random effects model was used for the meta-analysis if differences were observed in the study population or setting between studies. Otherwise a fixed effect model was applied. *p *value <0.05 was considered as statistically significant. Sensitivity analysis was carried out using the leave-one-out approach. Publication bias was not assessed visually using funnel plot because less than 10 studies included in the meta-analysis.

## RESULTS

**Overview of literature search**

**Fig. 1:**
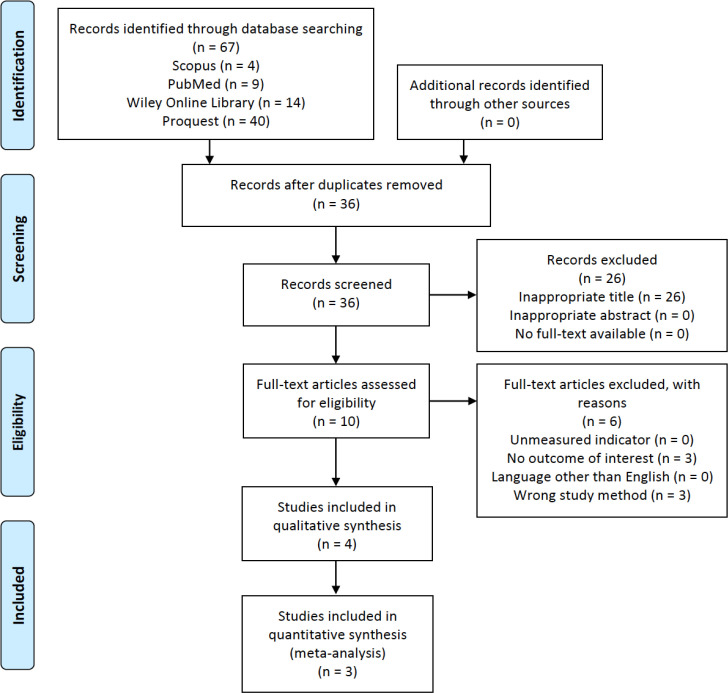
PRISMA flowchart of the study selection process^[12]^

The initial search of this study resulted in a total of 67 studies obtained from Scopus, PubMed, Wiley Online Library, and ProQuest. Of those, we screened 36 titles and abstracts after the removal of duplicates. Ten studies were further assessed based on the eligibility criteria. As a result, four studies were selected and then analyzed for qualitative synthesis, and three of four studies were analyzed for quantitative synthesis. One study by Awano *et al.*^[^^11^^]^ was excluded from the quantitative synthesis due to the data skewness from normality when the provided median and IQR data were transformed into mean and SD. The study selection process is provided in Figure 1.

**Characteristics and results of the selected studies**

A total of 151 adult male and female patients, 89 for non-severe COVID-19 and 62 for severe COVID-19 with a mean age of 58.98 ± 12.79, were successfully collected from four studies, comprising of two cohort studies^[^^10^^,^^11^^]^ and two cross-sectional studies^[^^15^^,^^16^^]^. The case definition of COVID-19 varies between studies. Nevertheless, patients in each study was generally divided into two main groups, the non-severe COVID-19 and the severe COVID-19 group. The KL-6 levels were higher in severe COVID-19 patients and were found to be statistically significant in all studies with *p* <0.05. Findings by two studies, d'Alessandro *et al.*^[^^16^^]^ and Awano *et al.*^[^^11^^]^, regarding the AUC, sensitivity, specificity, and best cut-off value of KL-6 levels were 82–84%, 76–83%, 86–89%, and 303–406.5 U/mL, respectively. More details on the included studies are summarized in Table 1. The quality assessment of each study using the NOS score is listed in Table 2. In the cohort studies, the NOS score showed 5 and 9 points on each study, indicating moderate and good-quality studies, while in the cross-sectional studies, the NOS score was 6 and 9, meaning satisfactory and very good studies, respectively.

**Meta-analysis of serum KL-6 in severe vs. non-severe COVID-19 patients**

**Fig. 2:**
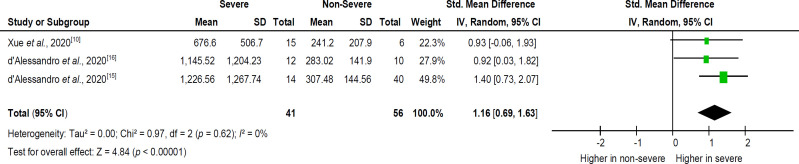
SMD of serum KL-6 levels in severe vs. non-severe COVID-19 patients

A pooled analysis from three^[^^10^^,^^15^^,^^16^^]^ out of four studies (Fig. 2) showed that KL-6 levels in severe COVID-19 patients were significantly higher (SMD = 1.16; 95% CI = 0.69–1.63) than non-severe COVID-19 patients. The heterogeneity of KL-6 levels between studies was considered low (*I*^2^ < 25%). We further observed differences in the case severity definition of COVID-19 between studies; therefore, a random effects model was applied.

**Table 1:** Basic characteristics and results of KL-6 findings of the included studies

**Author**	**Place**	**Study design**	Case Definition of COVID-19*
Xue *et al.*^[^^10^^]^	First Affiliated Hospital of Guangzhou Medical University	Prospective cohort study	Mild-common and severe-critically severe COVID-19 were referred based on the diagnostic and grading criteria of three sources. Corman VM, Landt O, Kaiser M. *et al.* Detection of 2019 novel coronavirus (2019-nCoV) by real-time RT-PCR. Euro Surveill. 2020; 25:2000045. Chu DKW, Pan Y, Cheng SMS, Hui KPY, Krishnan P, Liu Y, Ng DYM, Wan CKC, Yang P, Wang Q, Peiris M, Poon LLM. Molecular Diagnosis of a Novel Coronavirus (2019-nCoV) Causing an Outbreak of Pneumonia. Clin Chem. 2020; 66:549-555. Jin YH, Cai L, Cheng ZS. *et al.* A rapid advice guideline for the diagnosis and treatment of 2019 novel coronavirus (2019-nCoV) infected pneumonia (standard version). Mil Med Res. 2020; 7:4.
d'Alessandro, *et al.*^[^^15^^]^	COVID Unit of Siena University Hospital	Cross-sectional study (Retrospective)	Severe COVID-19 was defined as patients with the need for ICU admission, MV, or high-flow oxygen therapy. Otherwise, patients were classified as non-severe COVID-19.
d'Alessandro, *et al.*^[^^16^^]^	Siena University Hospital	Cross-sectional study (Prospective)	Severe COVID-19 was defined as patients who underwent intubation and MV in the COVID-19 ICU. Otherwise, patients were classified as non-severe COVID-19.
Awano *et al.*^[^^11^^]^	Japanese Red Cross Medical Center	Retrospective cohort study	Participants with the diagnosis of COVID-19 were categorized into mild-moderate and severe-critical groups with the following definitions: Mild COVID-19 was defined as patients who had any of the various signs and symptoms of COVID-19 without shortness of breath, dyspnea, or abnormal chest imaging. Moderate COVID-19 was defined as patients who had lower respiratory disease on clinical assessment or imaging and SpO_2_ ≥94% on room air at sea level. Severe COVID-19 was defined as patients who had a respiratory rate of >30 breaths/min, SpO_2_ <94% on room air at sea level, PaO_2_/FiO_2_ <300 mmHg, or lung infiltrates >50%. Critical COVID-19 was defined as patients who had respiratory failure, septic shock, and/or multiple organ dysfunction.

* Provided statements were directly collected from each study without any re-citations in this study. ICU, intensive care unit; MV, mechanical ventilation.

**Table:** Continue of Table 1.

Author	Population	Sample size (M/F)	Age of patients^†^	KL-6 levels (U/mL)		*p * value	AUC	Best cut-off value (U/mL)	Sn/Sp (%)
				Median (IQR)	Mean ± SD**				
**Xue ** ***et al.*** ^[^^10^^]^	Non-severe (mild & common) COVID-19	6(2/4)	55.00 ± 18.84	N/A	241.20 ± 207.90	< 0.01	N/A		
	Severe (severe & critically severe) COVID-19	15(12/3)	57.20 ± 14.25	N/A	676.60 ± 506.70				
**d'Alessandro ** ***et al.*** ^[^^15^^]^	Non-severe COVID-19	40(21/19)	64(58, 72)	316(210, 398)	307.48 ± 144.56	< 0.0001	N/A		
	Severe COVID-19	14(12/2)	65(59, 71)	1125(495, 2034)	1226.56 ± 1267.74				
**d'Alessandro ** ***et al.*** ^[^^16^^]^	Non-severe COVID-19	10(6/4)	64(51, 64)	293(197, 362)	283.02 ± 141.90	= 0.0118	82.4%(95% CI = 62-100; *p* = 0.0129)	406.5	83/89
	Severe COVID-19	12(9/3)	62(60, 68)	1021(473, 1909)	1145.52 ± 1204.23				
**Awano ** ***et al.*** ^[^^11^^]^	Non-severe COVID-19	33(23/10)	40(33, 50)	223(166, 255)	214.10 ± 68.97	< 0.001	84%	303	76.2/86.2
	Severe COVID-19	21(15/6)	64(56, 78)	338(303, 529)	N/A				

^†^Age of patients is presented as years in mean ± SD or median (IQR). ^**^All mean ± SD values were transformed from the median (IQR), except Xue *et al.*^[10]^. M, male; F, female; N/A, not available or not applicable; Sn, sensitivity; Sp, specificity

**Accuracy of serum KL-6 for predicting severe COVID-19**

**Fig. 3:**
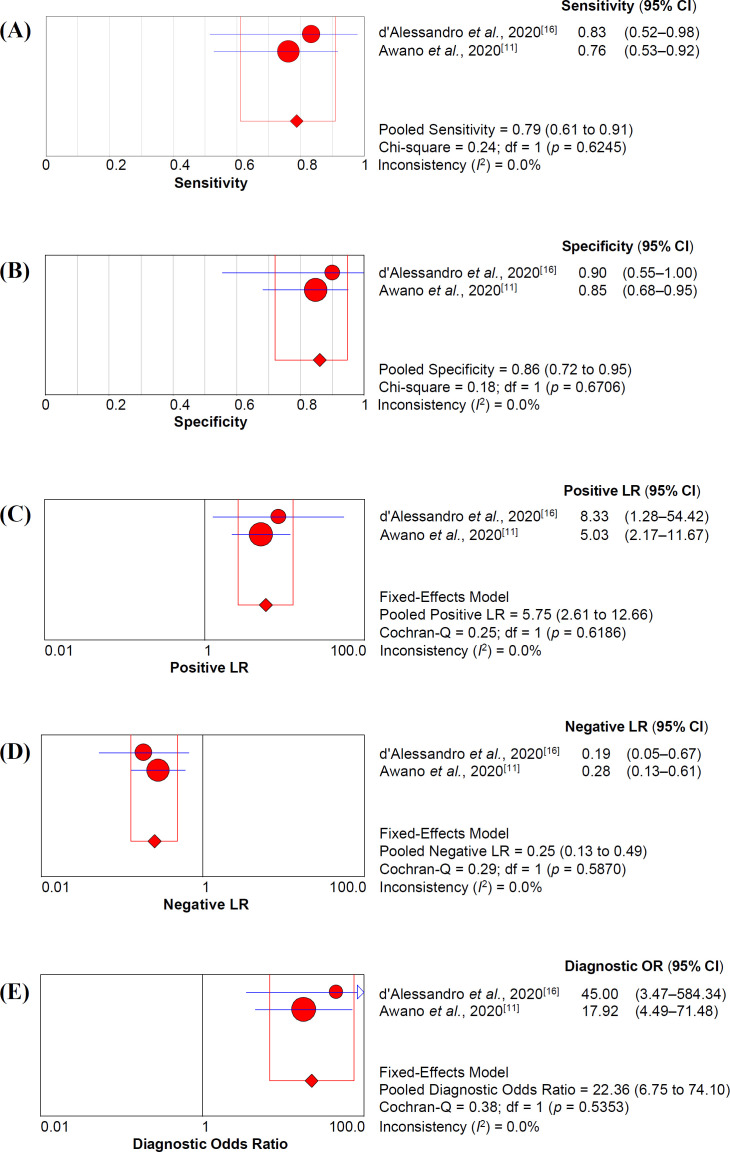
Pooled serum KL-6 accuracy analysis of sensitivity (A), specificity (B), positive likelihood ratio (C), negative likelihood ratio (LR), and diagnostic odds ratio (E) in predicting severe COVID-19

 Pooled accuracy analysis of serum KL-6 in two studies^[^^11^^,^^16^^]^ is depicted in Figure 3. The sensitivity and specificity of serum KL-6 were 79% (95% CI = 61–91%) and 86% (95% CI = 72–95%), respectively. Furthermore, +LR and –LR of serum KL-6 were 5.75 (95% CI = 2.61–12.66) and 0.25 (95% CI = 0.13–0.49), respectively. We also assessed diagnostic odds ratio with a value of 22.36 (95% CI = 6.75–74.10).

**Sensitivity analysis**

Sensitivity analysis was performed to assess the influence of each individual study on the pooled subgroup and overall results. The results of the sensitivity analyses suggested that the statistical significance of pooled subgroups and overall point estimates in all meta-analyses were not affected by any single study.

## DISCUSSION

To the best of our knowledge, this study was the first meta-analysis discussing serum KL-6 in COVID-19. Our study showed that serum KL-6 was a useful biomarker in predicting the severity of COVID-19 lung injury. In the current study, we further analyzed the pooled accuracy of serum KL-6 with moderately high sensitivity and specificity accompanied by moderate +LR and –LR. In such scenario, serum KL-6 can be a valuable test for predicting the needs of aggressive therapy when the pre-test probability of severe COVID-19 was uncertain (34–66%) or unlikely (10-33%). This method was based on Bayes's theorem^[^^17^^]^.

The latter findings indicated that serum KL-6 in COVID-19 cases was also a reliable and helpful biomarker in confirming the practitioners’ treatment decision.

KL-6 is a transmembrane mucin-like glycoprotein with a high molecular weight and is classified as human MUC1 mucin, encoded by the *MUC1 *gene. This mucoprotein is mainly produced by proliferating or damaged type II alveolar epithelial cell on its cell membrane. It is also expressed on the epithelial cells of bronchus, esophagus, stomach, pancreas, and normal basal cells of the terminal bronchiolar epithelium. Soluble KL-6 is released from the damaged cells into the surrounding tissues and subsequently entered the bloodstream^[^^18^^-^^22^^]^. As one of the systemic markers, plasma KL-6 level has been shown to be increased in patients with acute lung injury and ARDS^[^^23^^]^. Therefore, it has been suggested that the serum KL-6 level may potentially serve as a specific indicator for lung injury^[^^18^^,^^19^^]^. In addition, other studies have shown the potential of KL-6 as a prognostic and diagnostic tool in several interstitial lung diseases, such as sarcoidosis and idiopathic interstitial pneumonia^[^^8^^,^^24^^]^. KL-6 could easily reach the blood via disrupted alveolar-capillary barrier mainly. As a result, KL-6 would circulate through the blood vessels and act as a chemoattractant to fibroblasts. Several effects of KL-6 on lung fibroblasts as a pro-fibrotic and an anti-apoptotic have also been shown. This may explain the fibrosis event in the high level of serum KL-6^[^^22^^,^^25^^]^.

**Table 2:** Quality study assessments of cross-sectional and cohort studies using NOS score

NOS score of cross-sectional study				NOS score of cohort study		
Components	d'Alessandro*et al.*^[^^15^^]^	d'Alessandro*et al.*^[^^16^^]^		Components	Xue *et al.*^[^^10^^]^	Awano *et al.*^[^^11^^]^
Selection						
**Representativeness of the sample**	1	1		Representativeness of the exposed cohort	1	1
**Sample size**	1	1		Selection of the non-exposed cohort	1	0
**Non-respondents**	1	1		Ascertainment of exposure	1	1
**Ascertainment of exposure**	1	2		Demonstration that outcome of interest was not present at the start of the study	1	1
Comparability						
**Comparability of subjects in different outcome groups on the basis of design or analysis**	0	1		Comparability of cohorts on the basis of design or analysis	2	0

Exposure						
**Assessment of outcome**	1	2		Assessment of outcome	1	1
**Statistical test**	1	1		Enough follow-up time length for the outcome to occur Adequacy of follow-up of cohorts	1	0
					1	1
Study Quality						
**Total Score**	6	9			9	5
**Interpretation**	Satisfactory	Very Good			Good	Moderate

The SARS-CoV-2 infection would stimulate the body’s immune response. Consequently, the secretion of several immunoglobulins and chemical products occurs. Moreover, this infection would also disrupt the integrity of the epithelial/endothelial barrier and the lung capillary endothelial cells, including pneumocytes^[^^26^^]^. This destruction leads to the high release of KL-6 by the damaged pneumocytes, particularly the type II pneumocytes^[^^22^^]^. With further deterioration in COVID-19 patients, the amount of viral load will increase. The huge viral replication process impairs the alveolar epithelium. As a result, the KL-6 is secreted. The disruption in the alveolar epithelium causes leakage in the basement membrane, which later enhances the permeability of the lung vasculature, resulting in the increase of serum KL-6 level. Therefore, the level of serum KL-6 may represent the extent of lung damage in COVID-19 patients^[^^5^^,^^10^^]^. A case report of two COVID-19 patients by Nakamura *et al.*^[^^27^^]^ showed a difference in serum KL-6 level, which later both cases were defined as different primary “phenotypes” of COVID-19 patients, namely type L and H^[^^28^^]^. In type L patients, characterized by low elastance (high compliance), low ventilation to perfusion ratio, low lung weight, and low recruitability, the type II pneumocytes were relatively intact. Therefore, it resulted in a normal range of serum KL-6 level (131–363 U/mL) for 21 days. However, the type H patient characterized by high elastance, high right-to-left shunt, high lung weight, and high recruit ability,showed a significant increase of serum KL-6 level (673 to 2927 U/mL) for 21 days^[^^27^^,^^28^^]^.

This study has several limitations. First, the number of studies analyzed serum KL-6 in depicting COVID-19 severity and in its correlation with other clinical markers of COVID-19 is currently limited. Second, the number of participants involved in the included studies was also limited. Third, the influence of race on the serum KL-6 level was not assessed in our study due to the limitation of the study locations, which are only China, Italy, and Japan.

 In conclusion, high serum KL-6 might depict more severe lung injury in COVID-19 patients with moderately high sensitivity and specificity accompanied by a moderate positive likelihood ratio. However, further studies are still required to clarify and strengthen the current evidence in multi-centered studies with larger scales and more participants.

## CONFLICT OF INTEREST.

None declared.
